# Bridging the Chasm: Challenges, Opportunities, and Resources for Integrating a Dissemination and Implementation Science Curriculum into Medical Education

**DOI:** 10.1177/2382120518761875

**Published:** 2018-04-04

**Authors:** Tamar Ginossar, Carolyn J Heckman, Deborah Cragun, Lisa M Quintiliani, Enola K Proctor, David A Chambers, Ted Skolarus, Ross C Brownson

**Affiliations:** 1 Department of Communication & Journalism and the Comprehensive Cancer Center, The University of New Mexico, Albuquerque, NM, USA; 2 Cancer Prevention and Control Program, Fox Chase Cancer Center, Philadelphia, PA, USA; 3 Department of Global Health, College of Public Health, University of South Florida, Tampa, FL, USA; 4 School of Medicine, Section of General Internal Medicine, Boston University, Boston, MA, USA; 5 Brown School of Social Work, Washington University in St. Louis, St. Louis, MO, USA; 6 Division of Cancer Control and Population Sciences, National Cancer Institute, Bethesda, MD, USA; 7 Section Chief, Urology, VA Ann Arbor Healthcare System Associate Professor, Department of Urology, *University of Michigan* VA Ann Arbor HSR&D Center for Clinical Management Research.; 8 Prevention Research Center in St. Louis, Brown School, Washington University in St. Louis, St. Louis, MO, USA; 9 Division of Public Health Sciences and Alvin J. Siteman Cancer Center, Washington University School of Medicine, Washington University in St. Louis, St. Louis, MO, USA

**Keywords:** Dissemination and implementation, implementation science, knowledge transfer, medical education, systematic review and translational science

## Abstract

**Background:**

Physicians are charged with implementing evidence-based medicine, yet few are trained in the science of Dissemination and Implementation (D&I). In view of the potential of evidence-based training in D&I to help close the gap between research and practice, the goal of this review is to examine the importance of D&I training in medical education, describe challenges to implementing such training, and provide strategies and resources for building D&I capacity.

**Methods:**

We conducted (1) a systematic review to identify US-based D&I training efforts and (2) a critical review of additional literature to inform our evaluation of the challenges and opportunities of integrating D&I training in medical education.

**Results:**

Out of 269 unique articles reviewed, 11 described US-based D&I training. Although vibrant and diverse training opportunities exist, their capacity is limited, and they are not designed to meet physicians’ needs. Synthesis of relevant literature using a critical review approach identified challenges inherent to changing medical education, as well as challenges related to D&I science. Finally, selected strategies and resources are available for facilitating incorporation of D&I training into medical education and overcoming existing challenges.

**Conclusions:**

Integrating D&I training in the medical education curriculum, and particularly in residency and fellowship training, holds promise for bridging the chasm between scientific discoveries and improved patient care and outcomes. However, unique challenges should be addressed, including the need for greater evidence.

## Introduction

One of the key tasks facing medical education consists of training physicians who can bridge the translational gap between research and practice. With estimates that 1 out of 3 patients receive care that does not comply with current scientific evidence,^1^ the provision of evidence-based care is a key challenge.^2–4^ Medical educators are also grappling with this gap,^4–7^ and calls for reforms of medical education are at least a century old.^7^ For instance, following decades-long efforts to teach patient safety and quality improvements and identification of best practices,^8^ dissemination of such programs is lacking.^9^ Addressing translational gaps involves multifaceted, complex processes that consider the context as well as the systemic nature of adoption of innovations. Dissemination and implementation (D&I) science is tasked with identifying effective ways to reduce translational gaps between research and practice, often referred to as the “valley of death.”^10^ According to the National Institutes of Health (NIH), *dissemination* refers to the purposive distribution of health information and evidence-based interventions, whereas *implementation science* refers to the study of how to integrate research findings into evidence-based policy and practice.^11^ As a new science, however, consistency of concepts and their definitions remains a challenge, and knowledge translation, knowledge transfer, and diffusion, as well as similar constructs are related concepts that often include overlapping definitions.^12,13^

The NIH,^14^ Institute of Medicine (IOM),^15^ Veterans Administration, Agency for Healthcare Research and Quality,^16^ AcademyHealth, American Board of Internal Medicine, and Patient-Centered Outcomes Research Institute^17^ have all declared advancing D&I efforts to reduce the translational gap to be a priority area, which creates critical pressure on the next generation of clinicians and medical educators. Numerous authors emphasized the importance of building stakeholders’ capacity for D&I of interventions in medical education,^18–23^ health care,^24^ and community settings.^25^ In view of physicians’ key role as change agents and leaders in the health care system, including medical education,^26,27^ researchers and practitioners raised concerns about lack of training in the science of how to lead such changes^18,28^ and a growing number of medical education scholars underscored the importance of training physicians in D&I.^16,29–32^

Despite the recognized need to build physicians’ capacity in D&I, integration of D&I training into medical education and the specific needs and challenges of such integration have not been previously examined. Therefore, in this review, we aim to identify and describe specific D&I training opportunities and to critically examine literature on D&I training and medical education to identify challenges associated with possible integration of D&I training into medical education, as well as available resources. We acknowledge important D&I training programs in Canada.^33,34^ However, in view of the importance of the health care context and particular requirements of medical education in the United States, we focus this analysis on training opportunities available in the United States. Specifically, the objectives of this critical narrative review are 3-fold: (1) to examine the importance of D&I training in medicine and medical education nationally, (2) to describe challenges to implementing such training, and (3) to provide strategies and resources for building D&I capacity in medical education.

## Materials and Methods

In view of our focus on integration of D&I training in medical education curriculum, we selected a critical review approach.^35^ This approach aims to document a comprehensive search of the literature and to provide a critical evaluation of its content. Effectiveness of critical reviews is measured in the degree to which they present, analyze, and synthesize materials from diverse sources. This method provides an opportunity to assess the current situation based on a previous body of work and to propose a new path based on synthesis of different schools of thought.^35^

First, we used systematic literature review processes^36^ to identify all articles reporting on specific D&I training programs in the United States. We searched multiple databases (Medline, PubMed, CINAHL Complete, Web of Science, and EBSCOhost) in December 2016. Search terms included “dissemination and implementation training,” “D&I Training,” and “implementation training.” Two reviewers independently reviewed the titles and abstracts of the retrieved articles.

**Figure 1.**
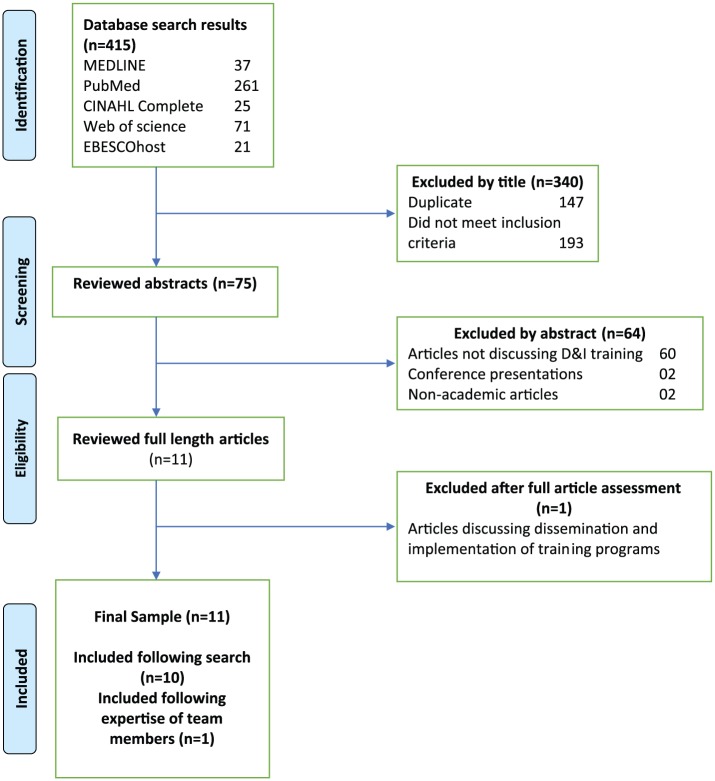
Flowchart of systematic literature review.

Inclusion criteria: articles reporting on specific training programs within the United States. Articles were excluded if they were not written in English or did not report on specific US-based D&I training opportunities. Following a critical review approach to literature synthesis,^37,38^ we also examined reference lists of widely cited papers and review articles. The identified articles were shared with the research team to ensure that no articles were overlooked (see Figure 1 for details on this process). Consistent with systematic review guidelines, 2 authors then independently evaluated the identified articles for strength of evidence regarding the evaluation and effectiveness of the D&I training.

Although the above systematic search strategies provide vigor, we also aimed at enhancing the scope of this review. In view of our overall goal of conducting a critical review that synthesizes different research disciplines and approaches, we also did a targeted literature search^35^ to locate articles that identified general factors associated with medical education curriculum changes. In contrast to systematic literature review, this search strategy is consistent with the goal of critical reviews “to collect, integrate and interpret results from the most compelling studies that satisfy the search terms and strategy. The search and written presentation need not be exhaustive.”^37,39^ Therefore, we judged the relevance and rigor of available research studies in relation to our overall focus, with the goal of summarizing findings from different studies qualitatively to inform our understanding of integrating D&I training in medical education. We therefore used 2 different search strategies to explore medical education as a context for D&I training that can explain challenges and opportunities to adopt D&I training.^39^

## Results and Discussion

### Opportunities for D&I trainings and outcomes

As illustrated in Figure 1, the systematic search yielded 415 articles, with 147 duplicates and 193 that on review of title did not meet eligibility criteria. We reviewed 75 abstracts, of which 10 articles described specific D&I training programs taking place in the United States.^40–49^ An additional article was identified by one of the authors,^50^ for a total of 11. Based on these articles and additional sources, we identified diverse D&I training formats, including webinars, conferences, training institutes, certificate programs, graduate courses and programs, internships, and fellowships. Selected programs and institutes are shown in Table 1.

**Table 1.** Summary of selected ongoing/recent US-based D&I training programs.

Program name	Goal(s)	Trainees	Length	Components	Outcomes	Funding source(s)	Citation
*Ongoing programs*							
Colorado Research in Implementation Science Program (CRISP) D&I workshop	To bring D&I training to local investigators	Investigators affiliated with CRISP and Colorado Clinical and Translational Sciences Institute (CCTSI), most with doctoral level training and limited D&I experience; University faculty (73%), research staff (8%), graduate students/fellows (15%), practicing health care providers (15%), other (13%); Trainee degrees included 36% MD, 38% PhD/DrPH, 35% MPH/MSPH/MS, 23% other	1.5-day workshop	1. Day 1: Introduction to D&I, strategies, evaluation and measurement, RE-AIM framework 2. Day 2: In-depth feedback on D&I proposals/projects, submission of short concept paper on D&I research, practice, or project management, group discussion	1. High overall satisfaction ratings 2. Increase in self-reported knowledge 3. Suggested areas of improvement included continued seminars and feedback 4. One-third of 6-month postworkshop evaluations reported: (a) A D&I scientific paper (b) A D&I grant proposal (c) New D&I collaborations	AHRQ, NIH/ NCATS Colorado CTSI; VA	Morrato et al^44^ (www.CRISPebooks.org)
Implementation Research Institute (IRI; http://iristl.org/)	1. To train implementation researchers 2. Production of scholarly implementation products (papers, books, and curriculum models)	10-11 PhD or MD research fellows per year, ranging from 1-year postterminal degree to full professor Fellow disciplines for 2010-2012 included psychology (61%), social work (13%), medicine (10%), psychiatry (6%), epidemiology (3%), and anthropology (6%)	Two annual week-long trainings with ongoing mentoring	1 Annual 5-day institute 2. Site visits 3. Mentoring monthly/bimonthly 4. Pilot research 5. Attendance at implementation science conference 6. Evaluations by fellows and faculty	1. Overall program satisfaction very high 2. Fellows from first 3 cohorts (n = 31) have received 52 funded awards 3. Average 7.64 publications submitted and/or published per fellow	NIMH (R25); VA	Stamatakis et al,^49^ Proctor et al^47^
Mentored Training for Dissemination and Implementation Research in Cancer (MT-DIRC; http://mtdirc.org/)	1. To focus on training for D&I research in cancer prevention and control 2. To develop and refine a set of core D&I competencies 3. To develop and refine a model curriculum specific to D&I research on cancer disparities	Postdoctoral fellows in the early stages of their careers, as well as mid-career scholars with an interest in D&I in the field of cancer	Two annual week-long trainings	1. One-week on-site training at Washington University in St. Louis 2. Monthly, long-distance, mentoring to help craft a competitive research proposal 3. Networking with other fellows and faculty to produce scholarly products 4. Pilot project funding	Several publications, presentations, and funded grant proposals, and the development and refinement of core competencies Social network analysis of patterns of mentoring and collaboration among IRI participants, modeling of the relationship between mentoring and subsequent collaboration	NCI (R25)	Padek et al,^46^ Luke et al^50^
Prevention and Control of Cancer: Post-Doctoral Training in Implementation Science (PRACCTIS; http://www.umassmed.edu/pracctis)		US citizens or permanent residents with recent doctoral degrees (MD, DO, PhD, etc) from a range of disciplines	2- or 3-year full-time postdoctoral program	1. Take specialized courses in implementation science 2. Complete optional Master of Science in Clinical Investigation 3. Receive team mentorship from faculty and site partners 4. Join research teams, gather data, publish manuscripts, 5. Participate in mock NIH grant review sessions and draw on career planning and placement resources	Not reported	National Cancer Institute	
UCSF Program in Implementation Science (https://accelerate.ucsf.edu/training/ids)	1. To design and implement effective interventions 2. To design comprehensive evaluations of interventions 3. To develop better grant proposals	Broad range of professionals engaged in D&I	A concentration track in a 2-year full-time master’s degree in clinical research or a 1-year part-time certificate program (http://ticr.ucsf.edu/courses/implementation_research.html)	1. Translating Evidence into Practice Theory and Design 2. Translating Practice into Evidence: Community-Engaged Research 3. Translating Evidence Into Practice: Individual-Centered Implementation Strategies 4. Program Evaluation in Clinical and Public Health Settings 5. Translating Evidence Into Practice: System-Centered Implementation Strategies 6. Translating Evidence Into Policy: Framing Research to Influence Policy	Not reported	UCSF	Gonzales et al^42^
Training in Dissemination and Implementation Research in Health 2016 (TIDIRH 2016; https://www.scgcorp.com/tidirh2016/index.html)	To encourage institutional interest in D&I by having participants share training methods with their home institutions	Junior or senior investigators with doctoral degree who have not previously received funding for D&I-related research Researchers in fields of medicine, behavioral medicine, nursing, medical anthropology, health economics, public health, or health policy	Three-month online course and a 2-day in-person workshop	1. Theory, implementation, and evaluation 2. Creating partnerships and multidisciplinary research teams 3. Research methods, design, and analysis 4. Clinical, community, and policy interventions	Not reported	NIH (OBSSR, NCI, NIDDK, NHLBI); VA	
*Recent programs*							
11 different programs in North America	Synthesis of multiple D&I research training programs	NA	Not reported	Review of existing programs	Lessons learned in mental health practice	NIH (NCI, NIDDK, NCATS)	Chambers et al^41^
Developing a field-based approach to D&I research training	To address key gaps and opportunities in D&I research training	NIH staff and 10 researchers	Not reported	NIH meeting addressing the overarching issue of developing D&I research training	1. Expert opinion on training 2. Identified resources and gaps	NIH	Proctor et al^51^
Implementation Research Workshop	To advance implementation research in respiratory, sleep, and critical care medicine	Researchers from diverse disciplines (n = 24) 15 MD (61%)	One day	Expert opinion on and experiences with barriers and facilitators to implementation	Recommendations for implementation were provided, but no assessment of the workshop	American Thoracic Society; NHLBI	Bender et al^40^
Training in Dissemination and Implementation Research in Health (TIDIRH)	1. To increase submission rate and quality of D&I grant applications and publications 2. To encourage institutional interest in D&I by having participants share training methods with their home institutions	35 investigators (new and experienced) with no prior major research funding Trainee disciplines include psychology (34%), medicine (17%), epidemiology (14%), health behavior/education (11%), other (24%)	Five-day training	1. Introduction, overview, design 2. Design and measurement 3. Intervention and methods 4. Scale-up 5. Evidence	1. Very high ratings of agenda relevance, appropriateness of teaching strategies, and confidence in applying skills 2. At 6-month follow-up, 72% of trainees had initiated a new D&I grant application 3. 28% of trainees received funding for a new D&I grant 4. 97% of trainees reported using TIDIRH skills/knowledge to influence thinking of colleagues 5. 33% participated in post-TIDIRH online networking platform; 67% participated in post-TIDIRH conference call	NIH (OBSSR, NCI, NIMH); VA	Meissner et al^43^
UAB School of Public Health D&I in Health Course	To complement and expand on existing D&I training programs	Current graduate public health students and academic researchers (24 students and 19 faculty total); faculty associated with School of Medicine (44%), School of Nursing (11%), College of Arts/Sciences (5%), School of Dentistry (5%), School of Health Professions (5%), School of Public Health (5%), and other (5%); Student degrees included bachelor’s (78%), master’s (18%), and medical degree (9%) and were associated with departments of health behavior (91%) and health care organization/policy (4%)	Biweekly 75-minute seminar over Fall semester (2012-2013)	1. Didactic lectures 2. Viewing/listening to online presentations 3. Reviewing pertinent resources 4. Collaborative learning project with mixed student/faculty teams	1. High overall satisfaction ratings 2. Suggested areas of improvement from students included logistics and delaying start of collaborative project to give time to learn general concepts; faculty recommended clearer expectations for collaborative project and opportunity to attend lectures	UAB	Norton^45^

Abbreviations: AHRQ, Agency for Healthcare Research and Quality; CIHR, Canadian Institutes of Health Research; EBP, evidence-based practice; KT, knowledge translation; NCATS, National Center for Advancing Translational Sciences; NHLBI, National Heart, Lung, and Blood Institute; NIDDK, National Institute of Diabetes and Digestive and Kidney Diseases; NIH, National Institutes of Health; NIMH, National Institute of Mental Health; OBSSR, Office of Behavioral and Social Sciences Research; UAB, University of Alabama at Birmingham; UCSF, University of California San Francisco; VA, Department of Veterans Affairs.

The 11 identified articles reported on 6 different D&I training programs, including the American Thoracic Society and National Heart, Lung, and Blood Institute Implementation Research (ATS-NHLBI IR) workshop that centered on implementation research in respiratory, sleep, and critical care medicine,^40^ 3 NIH-funded D&I training institutes,^41,43,46-50^ 2 university-based training opportunities,^42,44^ and a D&I in health course.^45^ Table 1 summarizes information about each of these training opportunities, including trainees, goals, assessment, and outcomes. Although most of the programs listed physicians among participants in the training,^43-45,48^ the authors did not report on any attempts to align curriculum or evaluation criteria to these trainees or to the mission of medical education.

Publications about these programs have focused on training needs, competencies, and frameworks.^42,46,48^ Trainees’ perceptions of these programs have generally been positive.^41,44,46,48^ Important factors in training satisfaction included the expertise of the faculty and trainees, faculty flexibility in adjusting content to meet trainee needs, highlighting concrete D&I examples,^42^ learning about the development of practice linkages,^45,49^ and enjoyment of collaborative learning projects.^45^ Faculty have reported challenges in deciding on the curriculum. These challenges related to striking a balance of didactics, focusing on structure versus interactivity and flexibility, and meeting the needs of trainees from different fields, institutions, and at various levels of career development.^43^

Unfortunately, the evidence regarding the effectiveness of these programs is limited. Only 5 of the 11 articles reported outcomes assessment.^43,44,48,50^ These outcomes included positive trainee perceptions of the programs^43,49,48-50^ and in some cases objective outcomes such as numbers of publications or grants awarded^43,47^ and networks formed.^50^ Timing of follow-up assessment differed, with 2 programs assessing outcomes at 6 months after the programs occurred^43,44^ and others looking cumulatively over several years and thus apparently at different time points after the training depending on the cohort.^47,49^ An additional limitation of the articles related to their strength of evidence. The studies reported were descriptive with no comparison groups. Findings of the 5 articles reporting on outcomes are therefore consistent with the fourth level of 5 acceptable levels assessing scientific evidence.^52^ Finally, although some outcomes reported were impressive, their relevance to medical education is not always clear. For example, 70% of grant proposals submitted by Implementation Research Institute trainees were funded.^47^ This outcome might be highly relevant to those engaged in research careers but the mission of medical education might require different measures of effectiveness.^5^

In summary, our analysis of the available literature on D&I US-based trainings reveals that the vibrant and diverse training opportunities described above provide exciting options for individuals interested in D&I, including physicians, yet the capacity of current training programs and their evidence base have not kept up with the growing demand for D&I workforce education and development.^43^ Consequently, the development and implementation of rigorous, sustainable training has been recognized as one of the major challenges facing the field of D&I.^41,48^ Despite the important role of physicians as change agents in the health system and congruence between the mission of medical education and D&I efforts, few if any, opportunities have been designed specifically for physicians. Moreover, scholars have not explored the importance and feasibility of consistently integrating D&I training into specific medical education training and the potential factors that should be addressed to facilitate such integration. To address this gap, we examine these factors in the following section.

### Barriers and facilitators to integration of D&I training into medical education

Medical education and practice patterns are complex and constantly evolving in response to scientific discoveries, technological advancement, social trends, and policy changes. The dynamic nature of practicing medicine poses challenges to medical education, including medical school curriculum, residency training, and fellowships.^53^ The diverse, systemic challenges facing training in different medical education contexts are well-documented.^25,54–56^ More than a dozen factors have been shown to be consistently associated with such changes.^55^ These factors relate to organizational culture, communicative factors such as internal networking, and factors within the external environment^55^ such as financial pressures.^56^ Integration of D&I into medical education programs necessitates addressing the above organizational factors and the pressures on medical curriculum, including competing agendas in an environment of limited time, financial resources, and faculty capacity.^56^

An additional challenge that should be addressed in transforming physicians’ behavior relates to considering not only the formal curriculum that resides in current medical school educational content but also the “hidden” curriculum, which relates to a less obvious, but more influential set of behaviors that should be recognized in attempts to change provider practices.^54^ Such changes are relevant on the continuum of medical education, including graduate medical training (ie, residency), just prior to setting providers free into the delivery system where they will face a host of organizational, provider and patient factors that may influence their behavior. The importance of graduate medical education is further underscored by recent scrutiny of the effectiveness of continuing medical education (CME). Although well-designed CME has been demonstrated to improve physician performance and patient outcomes,^57^ CME is often ineffective in changing medical practices.^58^

In addition to addressing challenges inherent to changing medical education as described above, integration of D&I training in medical education should address specific challenges inherent to the field of D&I. Such challenges include the difficulty in generalizing across delivery system contexts, defining and maintaining intervention fidelity, the extent to which adaptation of an intervention’s components influences effectiveness, as well as challenges related to funding availability and the timing of funding cycles.^59^ In addition, D&I is a transdisciplinary field, and its science and practice involve multiple and complex theories and models.^60^ Although this complexity increases the difficulty of implementing such training, it also increases its importance.^61^ In the following section, we discuss opportunities for overcoming the challenges in integrating D&I training in medical education.

### Strategies and resources to support improved implementation of D&I training into medical education

The prior sections provided examples of training programs that could inform D&I training within medical education and challenges to integrating D&I training into medical education that could be addressed. In this section, we present an overview of selected strategies and resources for facilitating incorporation of D&I training into academic medicine. We also highlight ways in which such strategies and resources can begin to address some of the inherent challenges in conducting D&I research and practice.

#### Understanding variability in contextual factors influencing medical curriculum change

Dissemination and Implementation calls attention to the important influences of various contextual factors, including differences in clinical settings, patient populations, and policies. All of these contextual factors are important to consider when trying to generalize study findings or implement evidence-based practices. Similarly, many factors are relevant to encouraging curriculum change and spurring innovative D&I training at the institutional level. Early on in the process, it is important to establish an organization’s level of readiness to adopt a curriculum change. In contrast to the recognition of the importance of identifying effective learning assessment strategies,^62^ organizational factors, including organizational readiness in medical education, are largely understudied.^63^ The Medical School’s Organizational Readiness for Curriculum Change is a validated questionnaire that provides a structured way to assess readiness.^64^ Furthermore, the need for change should be recognized among multiple levels and types of stakeholders and not be dictated by administration. Using a student-centered curriculum review team could be a strategy to solicit and apply student feedback into curriculum design in academic medicine.^65^ As described above, tailoring strategies to specific institutional contexts can be a challenge in conducting D&I research and practice. Institutionally supported mentorship programs could serve as a time-efficient strategy that is tailored to mentee needs^66^ either as an addition to D&I training or as a stand-alone D&I training program.^67^

#### Opportunities in graduate medical education for formalized D&I research and practice

Graduate medical education is uniquely positioned to adopt D&I training, as it provides “formal intersection of medical education and care delivery” according to the Association of American Medical Colleges (AAMC). The Accreditation Council for Graduate Medical Education (ACGME) employs best practices, research, and advancements across the continuum of medical education with a specific focus on 6 core competencies for residency and fellowship training including patient care, medical knowledge, interpersonal and communication skills, professionalism, practice-based learning and improvement, and systems-based practice.^26^ The latter is clarified as “Residents must demonstrate an awareness of and responsiveness to the larger context and system of health care, as well as the ability to call effectively on other resources in the system to provide optimal health care.” This core competency is very much aligned with opportunities for formalized D&I training during an influential growth period for early-career physicians. Moreover, residents and fellows are recommended to “participate in identifying system errors and implementing potential systems solutions” according to the ACGME’s common program requirements.^68^ However, challenges associated with the complexity of such efforts and the lack of D&I training may often lead to suboptimal efforts and lack of effective system solutions. Such potentially missed opportunities are evident in ACGME’s core competencies of systems-based practice and practice-based learning and improvement. Although residents are required to demonstrate patient safety and quality improvement skill, site visits indicated that many graduate medical education clinical learning environments “do not provide the necessary systems-based practice context for residents’ clinical experience.”^69^ The authors also expressed a concern about what they described as a potentially “lost opportunity to create a cadre of young physicians equipped to lead sustainable systems-based improvement in clinical care” (p. 991). Harnessing the enthusiasm of trainees and their fresh take on challenging delivery system dilemmas through formal D&I didactic and core competencies such as practice-based learning curriculum could be transformational for the next generation of practicing physicians and promote physician-scientists capable of not only advancing D&I science but also affecting population health through evidence-based implementation practices.

#### Organizational-level changes to promote the integration of D&I curricula

Internal D&I mentorship programs and incentivizing participation in national training programs (see Table 1) have the potential to advance D&I training in medical education. Such changes would require institutional-level changes, consistent with the recognized need to reorganize structural aspects of medical schools to promote health care innovation.^70^ Strategies for providing training and support to encourage budding physician-scientists interested in health systems innovation can be applied to spur demand from trainees to pursue areas of D&I science.^71^ We propose that these strategies include creation of career pathways and additional promotion criteria for those focused on D&I science that could be analogous to basic science which has a long tradition of integration into medical school core curriculum and a track record for promoting the physician-scientist model. Developing academic capacity in D&I could have tremendous spillover effects into the broader mission of the medical education system to improve population health. Moreover, as a way to limit the effects of departmental silos, a structure for blending (or “interweaving”) faculty from across departments into multiple curriculum committees can support a shared school mission^72^ including support for the multidisciplinary field of D&I science. Ranking and evaluating medical schools using metrics that value D&I research and practice outcomes (including metrics related to practice improvement and reduction in medical errors) has the potential to enhance institutional engagement and commitment to integrate D&I training into the core curriculum for medical students, residency training, and CME.

Advancing D&I research and practice as part of medical education can support the IOM vision of developing a “learning health care system,” designed to initiate and use the best evidence for the collaborative health care choices of each patient and provider by integrating the process of discovery as part of patient care.^73^ To encourage the transition toward this vision, D&I training would provide additional opportunities to support and increase the utility of the practice components of a medical school, including university hospitals and affiliated clinics as essential components in a learning health care system. To harness the benefits of a learning health care system, integrating D&I research and practice training into rotations, internships, residencies, and fellowships as a core component appears warranted. Fashioning clinician training as a D&I enterprise focused on iterative improvement of practice, implementation of evidence-based interventions, sustainability of high-quality care, and improved understanding of the interventions once they have been implemented can provide a unique way to advance the learning health care system and ultimately patient care.

## Conclusions

The chasm between biomedical discoveries and improved patient care has been deemed the “valley of death”^10^ as evidence-based practices and guidelines are not well adhered to. The literature that we have reviewed documented the state of the art in D&I science training. Despite the known challenges to changing the curriculum of medical education, existing training opportunities are not designed to meet the needs of medical education and are not ready to be disseminated and upscaled. Therefore, greater evidence is needed before such integration is viable. Based on this literature, we have provided suggestions for and examples of D&I training that could be incorporated into medical education. More rigorous research, including well-designed, targeted training efforts, is needed to successfully integrate D&I training best practices in medical education.
